# Valorization of Agro-Industrial Wastes by Ultrasound-Assisted Extraction as a Source of Proteins, Antioxidants and Cutin: A Cascade Approach

**DOI:** 10.3390/antiox11091739

**Published:** 2022-09-01

**Authors:** Cristina Mellinas, Ignacio Solaberrieta, Carlos Javier Pelegrín, Alfonso Jiménez, María Carmen Garrigós

**Affiliations:** Department of Analytical Chemistry, Nutrition & Food Sciences, University of Alicante, San Vicente del Raspeig, ES-03690 Alicante, Spain

**Keywords:** ultrasound-assisted extraction, agro-industrial peel wastes, active compounds, cascade extraction approach

## Abstract

The use of agro-industrial wastes to obtain compounds with a high added-value is increasing in the last few years in accordance with the circular economy concept. In this work, a cascade extraction approach was developed based on ultrasound-assisted extraction (UAE) for tomato, watermelon, and apple peel wastes. The protein and antioxidant compounds were obtained during the first extraction step (NaOH 3 wt.%, 98.6 W, 100% amplitude, 6.48 W/cm^2^, 6 min). The watermelon peels (WP) showed higher proteins and total phenolic contents (857 ± 1 mg BSA/g extract and 107.2 ± 0.2 mg GAE/100 g dm, respectively), whereas the highest antioxidant activity was obtained for apple peels (1559 ± 20 µmol TE/100 g dm, 1767 ± 5 µmol TE/100 g dm, and 902 ± 16 µmol TE/100 g dm for ABTS, FRAP and DPPH assays, respectively). The remaining residue obtained from the first extraction was subsequently extracted to obtain cutin (ethanol 40 wt.%, 58 W, 100% amplitude, 2 W/cm^2^, 17 min, 1/80 g/mL, pH 2.5). The morphological studies confirmed the great efficiency of UAE in damaging the vegetal cell walls. WP showed a higher non-hydrolysable cutin content (55 wt.% of the initial cutin). A different monomers’ profile was obtained for the cutin composition by GC-MS, with the cutin from tomato and apple peels being rich in polyhydroxy fatty acids whereas the cutin extracted from WP was mainly based on unsaturated fatty acids. All of the cutin samples showed an initial degradation temperature higher than 200 °C, presenting an excellent thermal stability. The strategy followed in this work has proved to be an effective valorization methodology with a high scaling-up potential for applications in the food, pharmaceutical, nutraceutical, cosmetics and biopolymer sectors.

## 1. Introduction

According to FAO, the world production of fruits and vegetables in 2019 reached 883 and 1128 million tons, respectively [1]. The fruit and vegetable processing industries generate large quantities of by-products, such as skins, seeds, pomace, leaves and peels, causing serious environmental, management and economic problems [2,3]. In addition, it has been estimated that the waste generated in the processing industry to get the final products to consumers can exceed 25% of the latter [4]. Thus, minimizing food waste by its transformation into high added-value compounds is an important milestone that has acquired great relevance in the so-called biorefineries, helping the environment, economy and society [5]. This strategy promotes the development of a “zero waste” bioeconomy by manufacturing economically viable products using waste as the raw material [6,7].

Tomato, watermelon and apple peel wastes have been mainly used as animal feed, without having an outstanding commercial value. Watermelon and apple are industrially processed for the preparation of juices, jams, sauces or salads; resulting in peel waste accounting for 35 wt.% of the raw material [8,9,10]. The by-products generated from tomato processing into juices, sauces or jams can represent up to 13% of the total production of this vegetable [11]. These peel by-products are known to be a source of several bioactive compounds such as proteins, polyphenols and cutin, which can be extracted resulting in an economic and environmental valorization approach [12,13].

Proteins are important macromolecules involved in the basic stages of fruit and vegetable growth. The protein content of apple, watermelon and tomato has been reported to be 0.20, 0.61 and 1.0–1.9 mg/100 g of edible portion (including peels), respectively [14]. The proteins from apple [15], tomato [16] and watermelon [17,18,19] seeds have been extracted by using conventional and non-conventional methods. Different bioactive compounds, such as lycopene, carotenoids, polyphenols, polysaccharides and sugars, have been co-extracted with proteins by other authors from tomato peel by-products using advanced extraction techniques, such as high-pressure homogenization (HPH), pulsed electric field (PEF) and supercritical CO_2_ [20,21,22]. Polyphenols are well-known for their high antioxidant activity and they are usually formed by aromatic rings with hydroxyl groups, organic acids and acylated sugars [23,24,25,26]. The polyphenols from tomato and watermelon peels were extracted by HPH [20], pressurized liquid extraction (PLE) [27], supercritical fluid extraction (SFE) [28] and cold maceration [29]; whereas microwave-assisted extraction (MAE) with ethanol aqueous solutions [30] or enzymes such as *Aspergillus* spp. [31] were used to obtain polyphenols from apple peels.

The cuticle of vegetables and fruits is composed of cutin (40–80 wt.%), which acts as a protective barrier preventing water loss and biological attacks. Cutin is a high molecular weight cross-linked biopolymer made of C16–C18 hydroxy fatty acids bound by ester bonds. This natural polymer has a high potential to obtain alternative materials to conventional plastics due to its excellent properties, such as gas barrier, thermal regulator, UV protection, flexibility and mechanical properties in the range of petroleum-based materials, biodegradability and hydrophobicity [11,13,32,33]. The complex chemical structure of cutin is mainly composed of monomers, such as 9(10),16-dihydroxyhexadecanoic acid, 16-hydroxy-hexadecanoic acid, 18-hydroxy-9,10-epoxyoctadecanoic acid and 9,10,18-trihydroxyoctadecanoic acid [32,34,35]. The cuticle thickness usually ranges between 0.02–200 μm and it is found in the deepest part of the cell wall, which makes the extraction process difficult [32]. Few studies related to cutin extraction have been reported in literature. Cutin was isolated from tomato peels by conventional alkaline hydrolysis extraction with aqueous NaOH solutions [13], hydrogen peroxide-assisted hydrolysis (NaOH/H_2_O_2_) [11] and hydrochloric acid-free precipitation of sodium carboxylate [36]. Cutin extraction from Golden Delicious and Red Delicious apples [37] and watermelon [9] peels by using enzymatic methods was also reported. In all of the cases, the proposed methods were time-consuming and involved using high temperatures and pressures. So, the use of more sustainable extraction processes should be investigated. The potential use of cutin-based renewable materials for packaging, biomedical, adhesive, lubricant and fragrance applications has been reported [32]. Recently, cutin valorization for the production of water-repellent flexible bio-based films from corn protein and tomato cutin were developed [38]. In another work, pectin/tomato cutin coatings were successfully used to mitigate the deterioration of harvested calamansi (*Citrofortunella microcarpa*) [39].

The extraction strategies from industrial wastes reported in the literature have been mainly based on conventional methods and individual target compound isolation. Alternatively, in a cascade biorefinery approach, a sequential extraction methodology stands out as obtaining several bioactive compounds simultaneously in the same process, reducing the use of raw materials, solvents and energy, thus contributing to an increase in the sustainability of the overall process [10,40,41]. Kehili et al. successfully applied a cascade approach for the extraction of carotenoids, proteins, hemicellulose and cellulose from tomato pomace by using three sequential extraction processes based on supercritical CO_2_, alkaline hydrolysis with NaOH and liquid hot-water hydrolysis, respectively [22]. Other authors evaluated theoretically a biorefinery scheme for the extraction of lycopene, cutin and pectin to obtain fuels from tomato pomace [42]. In another work, a cascade approach for the conversion of citrus peel waste into essential oils, pectin, fertilizers and succinic acid was employed, by using hydro-distillation, acid hydrolysis, precipitation and fermentation processes [43]. Dávila et al. successfully designed a biorefinery process for avocado residues to obtain phenolic compounds, ethanol, xylitol and oil, conducting an exhaustive environmental and technical–economic assessment. [44]. Other authors applied ultrasound-assisted dilute acid hydrolysis (UADAH) in a biorefinery approach for the production of essential oils, pectin and bacterial cellulose from citrus waste [45]. High hydrostatic pressure extraction (HHPE) and ultrasound-assisted extraction (UAE) were satisfactorily used for the sequential extraction of pectin and polyphenols from tomato peel waste [46].

UAE has received great attention in recent years due to its advantages over conventional extraction methods such as being simple, environmentally friendly, versatile, efficient and fast. Moreover, it has great potential to be applied in cascading approaches [47] due to its green impact on food compounds, such as enhanced extraction efficiency in active compounds present in fruits and vegetables (carotenoids [48], polyphenols [49,50,51,52], pectin [52,53] or lycopene [54]); less power and solvent usage; and low maintenance costs [55]. This technique has also proven to be scalable for industrial applications [56]. In this work, a biorefinery cascade approach for the valorization of tomato, apple and watermelon peel wastes to obtain proteins, polyphenols and cutin by UAE was proposed, for the first time. The protein and polyphenols extraction were firstly obtained followed by a sequential second extraction to isolate cutin, as an important raw biopolymer for obtaining bio-based materials with excellent water barrier properties. All of the fractions were evaluated in terms of extraction yield. In addition, the total protein content by the Bradford method, and total phenolic content (TPC) and antioxidant activity (ABTS, FRAP and DPPH assays) were determined for the proteins and polyphenols’ fractions, respectively. The obtained cutin was characterized by using structural (FTIR) and thermal (TGA, DSC) techniques, as well as main monomers’ identification and quantification by GC-MS. Finally, the effect of UAE on the microstructure surface of the waste samples was investigated by SEM.

## 2. Materials and Methods

### 2.1. Raw Materials and Reagents

The raw dried tomato (TP), apple (AP) and watermelon (WP) peels were provided by SSICA (Parma, Italy) and they were ground into powder form using a ZM 200 high-speed rotatory mill (Restch, Hann, Germany). The particles passing through a 0.5 mm sieve were used, without any further treatment, for proteins, antioxidants and cutin extraction. All of the chemicals were of analytical grade and they were purchased from Sigma-Aldrich (Madrid, Spain).

### 2.2. Ultrasound-Assisted Extraction (UAE)

**Figure 1:**
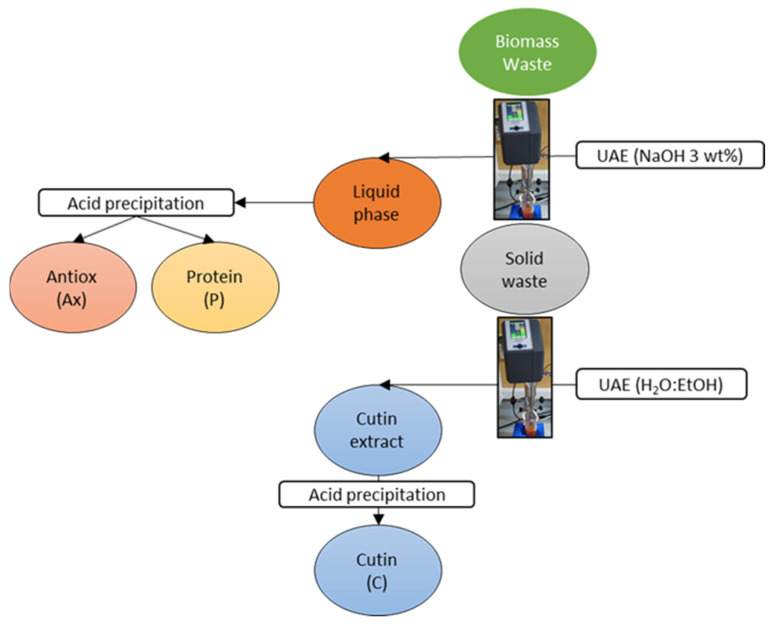
Scheme of the cascade approach process followed in this work for different biomass wastes.

A cascade approach based on UAE was followed to sequentially extract proteins, antioxidants and cutin (Figure 1). A UP400St Ultrasonic processor (Hielscher Ultrasonics, Teltow, Germany) equipped with a standard 524d22D sonotrode with a tip diameter of 22 mm was used. The solid waste amount (TP, AP, WP) and extraction solvent volume (NaOH 3 wt.%) were fixed to 2.0 g and 100 mL, respectively. The ultrasonic probe was immersed in a 150 mL beaker, containing the sample and solvent, and a chiller system was used for refrigeration. The temperature (60 °C) was kept constant during the extraction process to avoid the degradation of target compounds. According to previous experiments (data not shown), the extraction conditions used in this first step were 98.6 W, 100% amplitude, 6.48 W/cm^2^ and 6 min. The obtained extract was centrifuged to remove the raw material for 10 min at 5300 rpm using a Digicen 21R centrifuge (Ortoalresa, Madrid, Spain) and the recuperated solid was used in the following extraction process without any additional treatment. Next, HCl (3 M) was added to the liquid phase until reaching the isoelectronic point (pH 5–6, depending on the raw material), and the solution was centrifuged at 5000 rpm for 30 min. Then, the solid and supernatant composed mainly of proteins and antioxidant compounds, respectively, were recovered and frozen at −80 °C for 2 h. Finally, both products were lyophilized using a Telstar Lyoquest−55 PLUS (Terrassa, Barcelona, Spain) and stored under vacuum conditions until further analysis.

Cutin extraction was subsequently optimized by response surface methodology using a Plackett–Burman (III) design to obtain optimum extraction conditions from TP by UAE. Six factors were evaluated at different experimental conditions: EtOH content (40–100 wt.%), energy (40–60 kWs), amplitude (60–100%), solvent–sample ratio (40–80 mL/g), pH (2.5–5.5) and acid type (hydrochloric or citric acid). The optimized UAE conditions for TP were also applied to WP and AP samples for cutin extraction. A total of 1 g of solid material remaining after the first extraction was mixed with the adequate solvent proportion by UAE. The obtained extract was filtered to remove the raw material. Next, the acid solution was added at a fixed pH value and the solution was centrifuged at 5000 rpm for 30 min. The obtained solid was washed with distilled water and frozen at −80 °C for 2 h. Finally, the obtained cutin was lyophilized and stored under vacuum conditions until further analysis.

### 2.3. Characterization of the Obtained Fractions

Different analytical techniques were used to analyze the three fractions (proteins, antioxidant compounds, and cutin) obtained by UAE in order to evaluate their potential to be applied in different applications.

#### 2.3.1. Extraction Yield

The extraction yield of each recovered fraction (proteins, antioxidant compounds, and cutin) from all of the agro-food wastes was gravimetrically determined using the following equation:

Yield (wt.%) = 100 m_F_/m_W_ (1)

where m_F_ is the extract weight obtained after freeze-drying and m_W_ is the initial waste weight (TP, AP or WP).

#### 2.3.2. Total Proteins Content by the Bradford Method

The total soluble proteins content was evaluated by the Bradford assay for the recovered solid fraction (P) obtained after acid precipitation in the first extraction process. Then, 100 µL of extract and 4900 µL of Bradford dye were mixed in a polypropylene tube. After 5 min, the absorbance of the samples was read at 620 nm by using a Biomate 3 UV-vis spectrophotometer (Thermospectronic, Mobile, AL, USA). The standard curve was prepared by using bovine serum albumin as the protein standard [57,58,59].

#### 2.3.3. Total Phenolic Content (TPC)

Total phenolic content (TPC) was analyzed in the antioxidants fraction (Ax) obtained in the first extraction process. The TPC of the TP, WP and AP wastes was determined using the Folin–Ciocalteu assay according to Toor and Savage [60], with some modifications. Aliquots (0.5 mL) of each extract were mixed with 2.5 mL of Folin–Ciocalteu reagent, previously diluted in distilled water (1:10, *v*/*v*) and added with 2.0 mL of 7.5 wt.% aqueous sodium carbonate. Then, the mixture was vortexed and the absorbance was recorded at 765 nm after 30 min of incubation at 45 °C in the dark. Gallic acid in ethanol:water (60%, *v*/*v*) was used as the standard for quantification (5–80 mg/kg) (R^2^ = 0.9991) and the results were expressed as milligrams of gallic acid equivalents (GAE) per gram of dry waste. Each extract was analyzed in triplicate.

#### 2.3.4. Antioxidant Activity

The antioxidant activity of the Ax fractions obtained after the first extraction process for all of the wastes was evaluated by using three different assays (DPPH, ABTS, and FRAP). Each extract was analyzed in triplicate. The DPPH scavenging activity was determined as described by Szabo et al. [61]. Briefly, 0.4 mL of Ax was mixed with 2.1 mL of a freshly prepared DPPH solution (10^−4^ M in ethanol). The mixture was vortexed and incubated at room temperature in the dark for 120 min. Then, the absorbance was measured at 517 nm against a pure ethanol blank. Trolox in ethanol:water (60%, *v*/*v*) was used as the standard (5–70 mg/kg) (R^2^ = 0.9995). The results were expressed as milligrams of trolox equivalents (TE) per gram of dry waste.

The ABTS assay was performed according to Toor and Savage [60] with slight modifications. The ABTS radical cation was produced by mixing the ABTS solution (7 mM) with 2.45 mM potassium persulfate (1:1) and allowing the mixture to stand in the dark at room temperature for 12 h. The ABTS working solution was obtained by diluting with aqueous ethanol (60%, *v*/*v*) to a final absorbance of 0.70 ± 0.01 at 734 nm. Then, 0.3 mL of Ax was mixed with 3 mL of the ABTS working solution and the absorbance was measured after 120 min of incubation at room temperature in the darkness. Trolox in ethanol:water (60%, *v*/*v*) was used as the standard (5–60 mg/kg) (R^2^ = 0.9999). The results were expressed as milligrams of trolox equivalents (TE) per gram of dry waste.

The FRAP assay was determined according to Benzie and Strain [62]. The FRAP reagent was prepared by mixing 0.3 M acetate buffer (pH = 3.6), 10 mmol/L TPTZ made up in 40 mmol/L HCl and 20 mmol/L FeCl_3_ at a 10:1:1 ratio. Then, 0.1 mL of Ax was mixed with 3 mL of the freshly prepared FRAP reagent pre-heated at 37 °C. The mixture was vortexed and the absorbance was measured at 593 nm after 30 min of incubation at 37 °C. Trolox in ethanol:water (60%, *v*/*v*) was used as standard (5–100 mg/kg) (R^2^ = 0.9999). Results were expressed as milligrams of trolox equivalents (TE) per gram of dry waste.

#### 2.3.5. Scanning Electron Microscopy (SEM)

Morphological characterization of the raw waste materials (TP, AP, WP) and residues obtained after both of the sequential extraction processes was performed using scanning electron microscopy (SEM). The samples were coated with a gold layer under vacuum using a SCD 004 Balzers sputter coater (Bal Tec. AG, Furstentum, Lichtenstein) prior to analysis to increase their electrical conductivity. Microscopic images were elucidated using a JEOL JSM 840 scanning electron microscope (Peabody, MA, USA) at an accelerating voltage of 15 kV and different magnifications.

#### 2.3.6. Fourier Transform Infrared Spectroscopy (FTIR)

FTIR was used to confirm the presence of cutin during the optimization of UAE experimental conditions. In this sense, the extraction yield obtained during the second extraction step was only considered if obtaining a band around 1702 cm^−1^ in the FTIR spectrum. In any other case, the value given in the experimental design was 0. The cutin obtained using optimal extraction conditions was also analyzed by FTIR for all of the wastes. FTIR spectra of all of the samples were recorded with an infrared spectrophotometer Bruker Analitik IFS 66/S (Ettlingen, Germany) equipped with a KBr beam splitter, a deuterated triglycine sulphate (DTGS) detector and Bruker OPUS software (Version 3.1), also developed by Bruker Analitik. The analysis was performed, in triplicate, in the attenuated total reflectance (ATR) mode by using a Golden Gate accessory with a diamond crystal. The absorption spectra were obtained in the 4000–500 cm^−1^ range using 64 scans and a resolution of 4 cm^−1^.

#### 2.3.7. Gas Chromatography–Mass Spectrometry (GC-MS)

GC-MS was used to analyze the cutin composition obtained under optimal extraction conditions using TP, WP and AP samples. A total of 200 mg of each peel waste was stirred and refluxed with 25 mL of 0.1 M NaOCH_3_ in methanol for 2 h. The resulting solution was filtered using a 0.45 µm syringe filter. The obtained filtrate (pH 11–12) was taken to pH 6 using 0.2 M H_2_SO_4_, obtaining a brown precipitate which was assigned to the non-hydrolysable cutin. The solution was filtered to remove the brown precipitate and the obtained filtrate was evaporated in a rotary evaporator to remove the organic solvent. Then, a liquid–liquid extraction with 1:1 water-dichloromethane as the solvent was performed. The water phase was washed twice with CH_2_Cl_2_ and the accumulated organic phases were additionally washed twice with distilled H_2_O. Approximately 75 mL of the organic phase were obtained. The addition of 10 mL of saturated NaCl solution in each extraction of the aqueous phase was needed to achieve a good phase separation. The organic solvent was evaporated in a rotary evaporator to a few mL and then it was evaporated to dryness under a N_2_ current. A derivatization step was carried out by adding 0.125 mL of BSTFA (with 1 wt.% TMCS) in the presence of 0.125 mL of pyridine in test tubes at 60 °C for 15 min.

The identification of the main cutin monomers was performed by using an Agilent 7890N GC coupled to a 5977B quadrupole mass spectrometer (MS) (Agilent Technologies, Palo Alto, CA, USA), operating under the electronic impact (EI) ionization mode (70 eV). A HP-5MS capillary column (30 m × 0.25 mm × 0.25 µm) was used. The injector temperature was 300 °C and 1 μL of sample was injected. A flow rate of 1 mL/min of He (99.9%) was used as the carrier gas. The GC oven temperature program was 125 °C to 220 °C at 10 °C/min to 290 °C (maintained for 15 min) at 3 °C/min. The temperatures of ion source and quadrupole were 230 °C and 150 °C, respectively. The mass spectra were analyzed in the scanning mode (45–550 *m/z*). Agilent MSD ChemStation software was used for data analysis. The different compounds present in the samples were identified by matching the obtained fragmentation patterns with the National Institute of Standards and Technology (NIST) mass spectral database. The identification certainty between the sample spectrum and that identified by the library was given by the quality factor (%). Quantification was performed by using total ion peak areas and the composition of cutin monomers was expressed as relative peak area (%).

#### 2.3.8. Thermal Properties

The thermal properties of cutin obtained from the different wastes were evaluated by thermogravimetric analysis (TGA) and differential scanning calorimetry (DSC). TGA tests were performed, in triplicate, with a TGA/SDTA 851 Mettler Toledo thermal analyzer (Schwarzenbach, Switzerland). Approximately 6 mg of each sample was heated from 25 to 700 °C at 10 °C/min under nitrogen atmosphere (flow rate 50 mL/min). DSC tests were carried out to determine the glass transition temperature (T_g_) by using a TA DSC Q-2000 instrument (New Castle, DE, USA) under nitrogen atmosphere (flow rate 50 mL/min). Then, 4 mg of samples were initially submitted to −90 °C in isothermal mode for 3 min. The temperature program that followed consisted of a first heating from −90 to 150 °C, then cooling to −90 °C and a further second heating to 150 °C, all of the stages at 10 °C/min heating/cooling rate. Three replicates of each sample were performed.

### 2.4. Statistical Analysis

All of the experiments were performed in triplicate and the results are shown as mean value ± standard deviation (SD). Statgraphics Centurion XVI (Statistical Graphics, Rockville, MD, USA) was used to generate and analyze the results of the Placket–Burman III design. The graphic analysis of the main effects and interactions between the variables was used and the analysis of variance (ANOVA) was carried out. The differences between the values were assessed based on confidence intervals by using the Tukey test at a *p* ≤ 0.05 significance level.

## 3. Results

### 3.1. Proteins Extraction

The extraction yield obtained from the three analyzed wastes in the proteins fraction (Table 1) was significantly different (*p* < 0.05) and followed the increasing order AP < WP < TP. A high concentration of proteins was obtained in all of the cases, considering that seeds have been reported to possess higher proteins’ concentration compared to peels. The proteins reported from tomato seeds and peels were in the range of 23.6–40.9 wt.% [63,64] and 1.0–16.5 wt.% [63,65], respectively. Lu et al. also referred to the composition of tomato waste (peels, seeds, and pomace) and they concluded that the main valuable components present in the seeds and peels were largely different, being tomato peels rich in dietary fiber, lycopene, and phenols; whereas the seeds mainly consisted of oil and proteins [12]. Similar protein yields were obtained by other authors in TP (around 10 wt.%) [66,67] compared to that found in this work. A similar trend in proteins content was described for watermelon peels and seeds [18], with reported values for peels ranging 2.4–12.4 wt.% [68,69,70], in agreement with the yield obtained in our study. According to Hiamed et al., the chemical composition, including proteins’ content, of WP depends on several factors, such as variety, ripening and geographical localization [68]. Concerning apple peel proteins, the compositional values in the range of 2.8–8.1 wt.% [69,71] can be found in the literature, in close agreement with the values obtained in this work; although no specific studies dealing with proteins’ extraction in AP were reported due to their lower protein content compared to other compounds, such as pectin [72] or antioxidants [73].

UAE has been used to obtain proteins from different sources due to its high efficiency and potential scalability [74,75,76]. In UAE, ultrasonic waves produce acoustic cavitation, generating hotspots of high temperature and pressure, enabling the extraction of components present in plant cells. UAE is a rapid and cost-efficient technique which requires short times and has reduced hypothermal effects, since it does not require high temperatures to obtain high extraction yields, increasing the functional properties and reducing the degradation or denaturation of the extracted proteins [77].

The soluble proteins content of the obtained extracts was evaluated by using the Bradford method which is based on the binding of protein molecules to Coomassie Brilliant Blue G-250 dye under acidic conditions, resulting in a shift of maximum absorbance from 465 to 595 nm (color change from brown to blue) [58]. Significant (*p* < 0.05) differences between the obtained extracts in soluble proteins’ content were found (Table 1), which were related to the presence of the antioxidant compounds in the extracts as well as phenolic–protein binding, due to the ability of polyphenols to be associated with proteins [20]. The higher proteins content was obtained for WP (857 ± 1 mg BSA/g extract), in agreement with the results found by Solanki et al. [78] when comparing the composition of the primary metabolites from non-edible parts of several fruit wastes, including the peels of orange, sweet lime, watermelon, cucumber, pomegranate and mango; and the seeds from mango, custard apple, black plum and papaya.

The protein-rich extracts in waste peels can be also obtained by using polymer resins instead of isoelectronic point precipitation for purification [79]. The obtained extracts have shown great potential to be applied in different sectors, such as food packaging and functional foods for human or animal feed [80], due to their high soluble proteins content. The amino acids’ composition of the studied wastes has been widely reported. Low contents of methionine, leucine and tryptophan have been found in tomato by-products [12], whereas a high lysine content was obtained, which was similar to that found for other plant proteins [81]. The protein fraction of apple wastes is characterized by presenting high levels of aspartic acid [82]. Finally, watermelon protein contains adequate amounts of lysine, methionine, histidine, threonine and leucine, while valine and isoleucine are present in higher concentrations [18].

### 3.2. Antioxidants Extraction

#### 3.2.1. Extraction Yield of Antioxidants

Significant differences (*p* < 0.05) in the extraction yields (YAx, Table 1) between the antioxidant extracts obtained from the studied wastes were found. TP showed the highest YAx value (36 ± 5 wt.%), followed by AP (32 ± 3 wt.%) and WP (25 ± 6 wt.%). These differences may be due to variabilities in the vegetal structure, plasticity or compositional differences, which may lead to different ultrasound cavitation effects [83]. In addition, while TP and AP could be considered to have a similar plant structure, the WP stand out for their hardness. Previous studies on tomato, watermelon and apple by-products reported lower extraction yield values for antioxidant fractions compared to those obtained in the present work. Chada et al. obtained extraction yields of 16–24%, 6–7 wt.% and 15–17 wt.% by Soxhlet extraction, MAE and PLE, respectively, of antioxidant compounds from industrial tomato pomace [27]. An extraction yield of 1.63 wt.% was reported for polyphenols maceration with methanol [24] in watermelon by-products, which was much lower than the value obtained in our study. In another work, SFE was used and compared to Soxhlet extraction with ethanol and maceration in boiling water to recover phenolic compounds from apple pomace; with extraction yields ranging from 1–47 wt.%; showing the best results for Soxhlet extraction [28]. When comparing reported extraction yields in the literature, it should be considered that conventional extraction methods usually involve long extraction times and large solvent amounts in contrast to UAE which can increase the overall extraction yield of the active compounds present in agro-food wastes without having these disadvantages [84,85]. In addition, the methodology used in this work allows the sequential extraction of various active compounds, being a green, innovative and promising process for the recovery of polyphenols.

#### 3.2.2. Total Phenolic Content (TPC)

As secondary metabolites, phenolic compounds are present in plants, having great nutritional benefits for human health. These compounds are characterized by having more than one phenol group in their molecule. TP, WP and AP are good sources of phenolics, such as gallic acid, chlorogenic acid, *p*-coumaric acid, 3-hydroxybenzoic acid, apigenin-7-glycoside, isovanillic acid, (-)-epicatechin, naringenin, rutin or caffeic acid [23,86,87,88,89]. Table 2 shows the total phenolic content (TPC) found for the polyphenolic extracts obtained from tomato, watermelon and apple wastes. Significant differences (*p* < 0.05) were observed between TPC values which followed the increasing order AP < TP < WP.

The obtained TPC values for TP were comparable to those reported in literature. Coelho et al. obtained TPC values ranging from 11 to 192 mg GAE/100 g for the extraction of bioactive compounds from tomato skins and seeds by Ohmic heating (70 °C, 15 min, 70% ethanol) [90]. In another work, Fuentes et al. found a TPC value of 36.9 ± 0.8 mg GAE/100 g from TP in methanolic extracts by bath sonication for 5 min, which was lower than that obtained in the present work [91]. The extraction of phenolic compounds in tomato peels, seeds and wastes from different varieties was also evaluated, showing methanolic extracts obtained after sonication in a bath for 1 h (with vortex mixing every 10 min) similar TPC values (67–352 mg GAE/100 g) than in our study [92]. Navarro-González et al. studied the compositional analysis of commercial TP by following an extraction/hydrolysis procedure and they found a TPC value of 158.10 ± 7.70 mg GAE/100 g [86], similar to that obtained in this study. However, higher TPC results were reported by other authors by using different extraction techniques. HHPE, UAE and Soxhlet were used for the sequential extraction of pectin, polyphenols and fatty acids from TP wastes, reporting a TPC value of 1625.7 mg GAE/100 g for the polyphenols-rich fraction obtained from the samples previously depectinized and subjected to UAE (70% ethanol, 15 min) [46]. Similarly, SoLVE (solid–liquid multivariable extractor) and UAE (50% ethanol, 90 min) were used for the simultaneous extraction of carotenoids and polyphenols from industrial tomato waste (peels and seeds), obtaining TPC values of 652 ± 1 mg GAE/100 g and 380 ± 4 mg GAE/100 g, respectively [93]. Finally, Gharbi et al. evaluated various Tunisian tomato by-products, as a potential source of natural bioactive compounds, reporting TPC values of 12,046 ± 1271 and 8993 ± 1544 mg GAE/100 g for tomato peels and seeds, respectively [94].

The TPC value of WP was slightly higher than that obtained for TP (*p* < 0.05). Morais et al. reported TPC values of 75–147 mg GAE/100 g for the methanolic extracts obtained by maceration of WP, in line with the value obtained in our study [95]. In another work, the extracts obtained from watermelon rinds, peels, pulp and seeds by mechanical shaking at room temperature with methanol for 24 h were compared, showing peels with a TPC value of 8.7 ± 0.2 mg GAE/100 g [24], lower than that reported in this work. Higher TPC values were found by Kim et al. in hydrothermal extracts from watermelon green rinds obtained at 300 °C and 30 min (762.65 mg GAE/100 g) [96]. However, Fadimu et al. reported a maximum TPC value of 7.94 mg GAE/100 mL by UAE (47.82 °C, 31.63 min, 42.84% ethanol) from WP [97].

The TPC value obtained from AP was the lowest compared to the other studied wastes (*p* < 0.05). Similar TPC values were reported by Bai et al. from industrial apple pomace (62.68 ± 0.35 mg GAE/100 g) by MAE (650.4 W, 53.7 s, 62.1% ethanol) [98]. In another work, TPC values ranging from 126.42 to 239.22 mg GAE/100 g were found for extracts from fresh peels of seven apple varieties after ethanol extraction and centrifugation [99]. By using a similar extraction procedure, TPC values of 72–131 mg GAE/100 g were found from different apple genotypes [100], close to the values obtained in our study. Higher TPC results (2395 to 7096 mg GAE/100 g) were obtained by Ferrentino et al. by SFE at different experimental conditions for recovering phenolic compounds from apple pomace [28]. MAE with ethanol aqueous solutions was also applied to obtain polyphenols from apple peels and Red Delicious apple pomace showing higher TPC values (5040 mg GAE/100 g [30] and 1680 mg GAE/100 g [101], respectively). Finally, Blidi et al. reported a TPC value of 1932 mg GAE/100 g for ethanolic extracts from AP by conventional heating in an oil bath [102].

In conclusion, the results obtained for tomato, watermelon and apple wastes highlighted the efficiency of the cascade approach methodology followed in this study by UAE for obtaining polyphenol-rich extracts, considering also that NaOH 3 wt.% was used instead of an ethanol:water mixture which is considered a more favorable solvent for polyphenols’ isolation. The obtained extracts have potential applications in several industries such as food, cosmetics, textiles or packaging, where antioxidant, anti-inflammatory and/or antimicrobial properties may be required [103].

#### 3.2.3. Antioxidant Activity

Table 2 shows the results found for polyphenolic extracts obtained from tomato, watermelon and apple wastes in terms of antioxidant activity by ABTS, FRAP and DPPH assays. Significant differences (*p* < 0.05) were observed between all of the studied extracts tested by ABTS and FRAP, whereas similar DPPH values (*p* > 0.05) were obtained for TP and WP extracts which were significantly different compared to AP (*p* < 0.05). In all of the cases, significant (*p* < 0.05) higher antioxidant values were obtained for AP, highlighting the antioxidant potential of this waste source.

Regarding TP, values of 279 ± 3, 264 ± 2 and 159 ± 1 µmol TE/100 g for ABTS, FRAP and DPPH assays, respectively, were obtained. Similar DPPH values were reported for the methanolic extracts obtained by UAE from the peels of 10 different tomato varieties, ranging from 120 ± 2 and 255 ± 3 μmol TE/100 g [61]. Fuentes et al. reported FRAP and DPPH values of 46.9 ± 0.9 μmol Fe^+2^/g and 97.4 ± 0.2%, respectively, for the antioxidant activity of TP [91]. The antioxidant activity of methanolic extracts obtained from different tomato wastes by sonication in an ultrasonic bath with vortex intervals was also in line with the results found in our study (35–406 μmol TE/100 g and 51–181 μmol TE/100 g for ABTS and DPPH, respectively) [92]. However, lower ABTS values were reported for commercial TP by using an extraction/hydrolysis process (0.39 ± 0.05 μmol TE/100 g) [86], and industrial tomato waste extracted by SoLVE (0.0109 μmol TE/100 g) or UAE (0.0057 μmol TE/100 g) for the simultaneous extraction of carotenoids and polyphenols [93]. Managbanag Salas et al. evaluated the antioxidant capacity of three different tomato varieties by DPPH after percolation overnight using different solvents (distilled water, ethanol, acetic acid), resulting in a maximum value of 265 μmol TE/100 g [104]. The antioxidant activity of Tunisian tomato peel and seed by-products was lower than 50% in terms of DPPH radical scavenging [94]. In another work, a high antioxidant activity was found for antioxidant compounds and lycopene extracted from industrial tomato pomace with ethanol:ethyl acetate 50:50 (*v*/*v*) by PLE at 90 °C (2144 ± 42 μmol TE/100 g and 2655 ± 56 μmol TE/100 g for DPPH and FRAP, respectively) [27].

WP showed higher antioxidant activity in terms of ABTS and FRAP results (356 ± 1 µmol TE/100 g and 507 ± 4 µmol TE/100 g, respectively) compared to TP; although comparable DPPH results (*p* > 0.05) were obtained for both peel wastes (Table 2). Morais et al. reported FRAP values ranging from 10.33 to 16.68 µmol Fe^+2^/g for WP [95], whereas 104.04% and 95.16% were reported for ABTS and DPPH inhibition, respectively, in hydrothermal watermelon extracts by Kim et al. [96]. A high antioxidant capacity was found from watermelon skin extracts obtained by maceration with methanol (91.46 ± 3.45% and 55.75 ± 2.44% for ABTS and DPPH inhibition, respectively) [24]. In another work, Saad et al. reported 57% of DPPH radical scavenging and a FRAP value of 50 µmol Fe^+2^/g for WP extracts [29]. A high antioxidant activity was also found for UAE hydroethanolic extracts from WP (85.15% of DPPH inhibition) [97].

The highest antioxidant activity was obtained for AP by all of the tested methods (1559 ± 20 µmol TE/100 g, 1767 ± 5 µmol TE/100 g and 902 ± 16 µmolTE/100 g for ABTS, FRAP and DPPH, respectively), although a lower TPC value was shown. This behavior may be due to the co-extraction of other compounds with antioxidant capacity at experimental conditions, such as carotenoids [105] or pectin [72,106]. Ferrentino et al. applied different extraction techniques for antioxidants’ recovery from apple pomace, reporting DPPH values of 2249 ± 120 µmol TE/100 g by SFE (30 MPa, 45 °C, 2 h, 5% ethanol as co-solvent), 819 ± 84 µmol TE/100 g by Soxhlet extraction (6 h, ethanol) and 456 ± 4 µmol TE/100 g by boiling water maceration (100 °C, 37 min) [28]; with the results obtained in our work in the DPPH assay being higher than those found for conventional extraction techniques with their consequent well-known disadvantages. In another study, different apple genotypes were evaluated after centrifugation with ethanol, reporting lower DPPH values (59.1–122.3 µmol TE/100 g) compared to those obtained in our work [100]. A high DPPH radical inhibition of 93.7% was found for the MAE extracts (35 W, 149 s, 10.3 mL of 60% ethanol) obtained from apple pomace [101]. A high antioxidant capacity was also reported in methanolic extracts from AP by using ultrasounds for 90 min in the dark (3724–5936 µmol TE/100 g, 8470–16,321 µmol TE/100 g and 17,498–30,269 µmol TE/100 g for DPPH, ABTS and FRAP, respectively) [89]. Finally, Blidi et al. reported a FRAP value of 123.0 μmol AAE/g for ethanolic extracts from AP by conventional heating [102].

Based on the results obtained for FRAP, ABTS and DPPH assays, it can be concluded that the extracts from TP, WP and AP obtained by the cascade approach followed in this study have shown considerable antioxidant capacity; in particular, for AP. However, a direct correlation between TPC and antioxidant activity values was not found, as the extracts obtained from AP showed lower TPC values, but higher antioxidant activity compared to the other studied wastes. This behavior may be due to the co-extraction of other compounds with antioxidant capacity by using aqueous NaOH as a solvent, such as proteins [12] or lignin and hemicellulose [107].

### 3.3. Cutin Extraction

**Figure 2:**
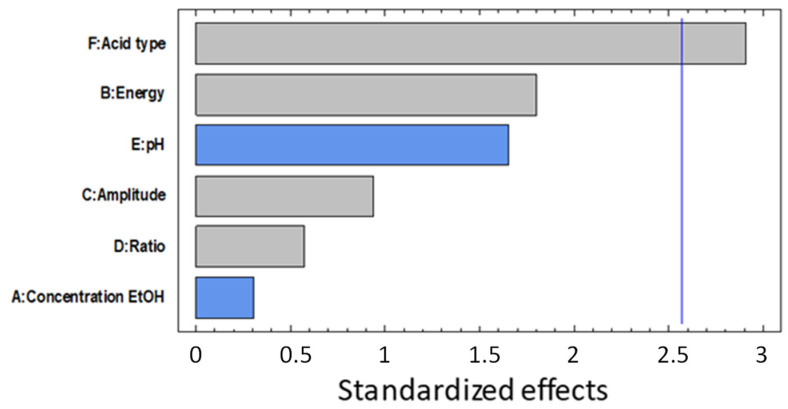
Pareto diagram of standardized effects in UAE for TP to obtain cutin.

The optimization of cutin extraction by UAE was performed using TP as the reference material. Table 3 shows the results obtained by using a Plackett–Burman (III) experimental design, where six different extraction variables were studied. The experimental conditions used in runs 3 and 10 did not show the characteristic FTIR cutin band appearing between 1702–1075 cm^−1^, and so, it was considered that no cutin was obtained. The best extraction yield (14.3 wt.%) was found using 40 wt.% ethanol, 60 kWs of energy, 100% of amplitude, and 1/80 g/mL at pH 2.5. Increasing the amplitude of the ultrasound waves also results in an increase in the cavitation phenomenon. Besides, a decrease in the solid/liquid ratio decreases the viscosity of the sample, allowing the ultrasound propagation to be more effective [54,83,108]. The effect of the different factors affecting UAE was determined using a Pareto diagram generated from the experimental design (Figure 2). The acid type (hydrochloric or citric acid) used showed a significant effect (*p* < 0.05) on cutin recovery. Moreover, the use of citric acid followed the principles of “green chemistry”, also improving the extraction yield of cutin. However, the other studied factors did not show a significant effect (*p* > 0.05) under the evaluated UAE conditions. These results were related to a high efficiency obtained in the first extraction process for proteins’ and antioxidants’ isolation.

**Figure 3:**
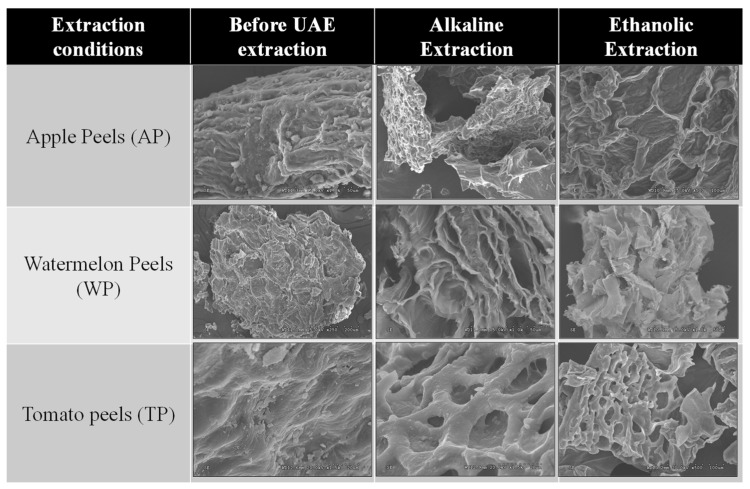
SEM images obtained for the different studied wastes before and after the UAE processes.

The effect of UAE on the microstructure surface of the waste samples was investigated by SEM (Figure 3). A significant change in the surface texture was observed for the residual samples obtained after UAE under alkaline conditions with cell-wall damage, confirming the great efficiency of this first step. The residues obtained after ethanolic extraction (second step for cutin extraction) presented some holes in the structure of the three studied wastes due to the effect of the ultrasound waves and the cavitation phenomenon. These results confirmed that the UAE cascade approach proposed in this work could promote a greater degradation of the cell walls, favoring the release of bioactive compounds from the studied agro-industrial wastes. As a consequence of this high cell-wall degradation, no significant effects (*p* > 0.05) were found for the UAE studied factors, except acid type, and the conditions used to obtain the highest extraction yield were considered to be the most adequate to obtain cutin from different peel sources.

#### 3.3.1. Extraction Yield of Cutin

The extraction yield of cutin in WP and AP (YC, Table 1) was determined by applying the optimal extraction conditions previously obtained for TP. Significant differences (*p* < 0.05) were found between the studied peel wastes, obtaining the highest value for AP (20 ± 1 wt.%). To the best of our knowledge, no previous research has been reported on determining the cutin extraction yields for AP and WP, although different works studying the complex composition of this biopolymer can be found [9,32,37]. On the contrary, the cutin obtained from TP was already studied by other authors [11,36,109]. Cifarelli et al. [36] obtained cutin from TP by alkali extraction, reporting extraction yields in the range of 4–28 wt.%, obtaining the best results using conventional heating for 2 h at 130 °C. In contrast, the use of 3 wt.% NaOH + 4 wt.% H_2_O_2_, under the same extraction conditions, showed the lowest cutin yield. Manrich et al. [35] developed a hydrophobic edible film based on cutin from TP which showed excellent barrier properties. These authors used a 3 wt.% NaOH solution (pH 14) followed by autoclaving at 121 °C for 120 min to obtain cutin. The obtained liquid phase was collected by filtration and then acidified with a 6 M HCl solution until pH 5–6 to precipitate cutin, reporting an extraction yield of 25 ± 2 wt.%. However, although these methods for cutin isolation in TP reported higher extraction yields, it should be considered that the applied experimental conditions involved the use of long times and high temperatures in contrast to the methodology used in our work, which greatly reduces extraction time and solvent consumption compared to conventional techniques. Moreover, the proposed approach has been demonstrated to be able to be applied to different peel wastes.

#### 3.3.2. FTIR Analysis

**Figure 4:**
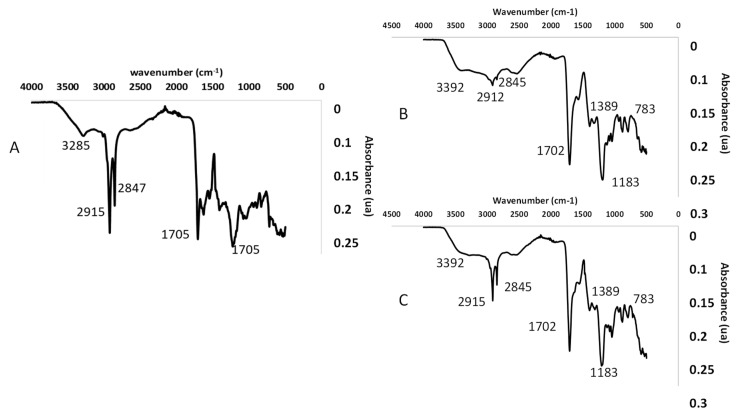
FTIR spectra of UAE cutin samples from tomato (**A**) watermelon (**B**) and apple (**C**) peels.

The FTIR spectrum of cutin was characterized by different bands corresponding to a polyester formed by polyhydroxy fatty acids. FTIR results confirmed the presence of cutin in UAE extracts according to the main characteristic band appearing at 1702–1705 cm^−1^ (Figure 4), which is associated with the carbonyl stretching of carboxylic acid ν(C=O) [11]. Based on the reported literature [13,35,36,110], the identification of characteristic bands of hydroxy acids were detected in the FTIR spectrum. The broad band observed at approximately 3282 cm^−1^ was associated with the stretching vibration of the hydroxyl group, while the bands in the 3000–2800 cm^−1^ region were assigned to -CH absorption including CH, CH_2_ and CH_3_ stretching vibrations. Other bands ascribed to the ester group were νa(C-O-C) at 1165 cm^−1^, vs(C-O-C) at 1116 cm^−1^, δ(CH_2_) at 1463 cm^−1^ and CH_2_ bending at 722 cm^−1^, indicating the presence of long-chain compounds. In addition, the FTIR spectrum of the cutin extracts showed different bands associated with aromatic compounds, such as ν(C=C) of phenolic acids (1632 cm^−1^), ν(C-C) aromatic (1605 cm^−1^) and (C-H) and (C-C) out of plane bending (835 cm^−1^). Finally, the two bands observed at 1054 and 1076 cm^−1^ were associated with bending of the hydroxyl (O-H) primary and secondary or stretching ν(C-O) of the polysaccharides present in the sample. In conclusion, FTIR results indicated that the cutin samples obtained by UAE from all of the studied wastes were mainly composed of long-chain hydroxy acids, phenolic compounds and some polysaccharides derived from non-hydrolysable cutin. The main differences observed between the FTIR results derived from the different studied cutin sources were related to their natural composition; mainly the fatty acids’ profile, which will be discussed in the following section.

#### 3.3.3. GC-MS Analysis

The non-hydrolysable cutin obtained from the studied wastes, expressed as a percentage of the initial cutin, is shown in Table 1. This non-hydrolysable solid is mainly composed of lignin and polysaccharides such as pectin, hemicellulose, cellulose and ash (mainly composed of macro-elements such as Ca, K, P and S, and micro-elements such as Zn, Cu, Fe and Mn) [13,111]. In particular, the tomato cuticle is known to consist of pectin and hemicelluloses (enriched in xyloglucan), as well as having a considerable crystalline cellulose content [112]. In some of the fruit cuticles, such as in watermelon, an additional resistant not hydrolysable lipid matrix may appear, called cutan [9], which remains after cuticle extraction and hydrolysis and presents a highly aliphatic character with a small proportion of aromatic moieties, increasing stiffness and strength [113]. A significant (*p* < 0.05) higher non-hydrolysable cutin content was found for WP (55 ± 9 wt.%) compared to the other studied wastes. Benítez et al. reported a value of 32 wt.% of dry pomace for non-hydrolysable cutin from TP after alkaline hydrolysis [13]. The percentages obtained for the non-hydrolysable and soluble cutins depend on the hydrolysis time and temperature. Non-hydrolysable cutin contents ranging from 5–10 wt.% were reported from different AP varieties subjected to alkaline hydrolysis for 8 h [37].

The complex mixture of monomers forming the cutin structure was identified by GC-MS analysis. The results obtained for the composition of cutin samples from TP, WP and AP are shown in Table 4. In general terms, the values obtained in this work by using a cascade approach from different sources were comparable with those reported in literature (Table 5). It is well known that the monomeric composition of the cuticular structure of fruits and vegetables is generated by a complex metabolic network which depends on many factors, such as plant type, growth and development; environmental stresses; or post-harvest life [33]. Typically, the monomeric cutin composition of fruits and vegetables consists mainly of polyhydroxy acids (abundance 16–92 wt.%). This monomeric variation in cutin composition directly affects its properties, such as gas and water barrier, biodegradability, mechanical, thermal, and hydrodynamic, protection against pathogens or UV damage, etc. These properties are closely related to the potential of cutin-based materials for packaging applications [32].

Cutin obtained from TP showed the presence of 10,16-dihydroxyhexadecanoic acid as the major compound (59.00 ± 7.02 wt.%). Higher contents were reported by Heredia-Guerrero et al. in cutin extracted from tomato fruit (81.6 wt.%) [32] and Cifarelli et al. for cutin extracted from TP (62 to 82 wt.%) [11]. In addition, Benítez et al. found dihydroxylated C16 as the predominant acid in cutin from tomato pomace, with a main content of 10,16-dihydroxyhexadecanoic acid of ~43 wt.%, lower than the value obtained in our work [13]. The 10,16-dihydroxyhexadecanoic acid is also present in the cutin from other plants and fruits, such as grapefruit (21.86 wt.%) and lime (40.36 wt.%) [114], or in leaves (17 wt.%), stems (10 wt.%) and flowers (47 wt.%) of Camelina sativa [115]. Several methyl and/or TMS derivatives found in our work, especially hydroxy acids, were also identified in previous studies [11,13,32]. Hexadecanoic acid (13.02 ± 2.05 wt.%), (9Z,12Z)-octadeca-9,12-dienoic acid (9.01 ± 2.02 wt.%), (Z)-octadec-9-enoic acid (5.02 ± 1.00 wt.%) and octadecanoic acid (3.00 ± 1.10 wt.%) were also identified in TP as major monomers (Table 4). These compounds appear to a considerable extent in both pomace and tomato seeds. In particular, a great abundance of (9Z,12Z)-octadeca-9,12-dienoic acid in tomato seeds (52.9 wt.%) was reported. The presence of both saturated (C14, C16 and C18) and unsaturated acids (9(en)-C18 and 9,12(dien)-C18) is also abundant in the monomeric composition of fruits and vegetables; and in particular, in tomato [11,32].

The fatty acids’ profile obtained for cutin from WP is also shown in Table 4. Several methyl and/or TMS derivatives were also identified by other authors using watermelon peels as a raw material to obtain cutin [9,32]. The main detected compounds were hexadecanoic acid (43.00 ± 1.02 wt.%), followed by (9Z,12Z,15Z)-octadeca-9,12,15-trienoic acid (16.00 ± 0.52 wt.%), (9Z,12Z)-octadeca-9,12-dienoic acid (11.10 ± 0.41 wt.%) and octadecanoic acid (12.02 ± 1.02 wt.%). In addition, 9,10-dihydroxyoctadecanedioic acid was also found in a lower content (2.51 ± 0.60 wt.%). The presence of these fatty acids in cutin from watermelon seeds and peels was reported by several authors. Regarding watermelon seeds, Eke et al. found a high (9Z,12Z)-octadeca-9,12-dienoic acid content (52.32 wt.%) whereas a lower hexadecanoic acid content (21.23 wt.%) was obtained [116]. Petchsomrit et al. reported hexadecanoic acid and (9Z,12Z)-octadeca-9,12-dienoic acid contents in watermelon seeds of 12.08 wt.% and 60.10 wt.%, respectively, as major components. Concerning WP, these authors found a lower hexadecanoic acid (28.42 wt.%) concentration and similar (9Z,12Z)-octadeca-9,12-dienoic acid (15.86 wt.%) content compared to those obtained in our study [117]. In another work, (9Z,12Z,15Z)-octadeca-9,12,15-trienoic acid and 9,10-dihydroxyoctadecanedioic acid were also found in WP in contents of 25.24 wt.% and 15.34 wt.%, respectively, higher than those obtained in this research [9].

The fatty acids profile obtained for cutin from AP (Table 4) showed (9Z,12Z)-octadeca-9,12-dienoic acid (20.02 ± 1.23 wt.%), (E)-octadec-9-enoic acid (12.17 ± 0.49 wt.%) and 9,10,18-trihydroxyoctadecanoic acid (10.53 ± 0.61 wt.%) as the main monomers. Several authors also determined the presence of these fatty acids in both the apple seeds and peels, with (9Z,12Z)-octadeca-9,12-dienoic acid being reported as the main compound present, in line with the results found in our work. (Z)-octadec-9-enoic acid and hexadecanoic acid were also found as the major components in apple seeds. Bada et al. reported contents of 50–60 wt.% for (9Z,12Z)-octadeca-9,12-dienoic acid, 27–37 wt.% for (Z)-octadec-9-enoic acid and 8–9 wt.% for hexadecanoic acid in different apple seed oils [118]. In another work, (9Z,12Z)-octadeca-9,12-dienoic acid was also reported in a high content (59 wt.%), followed by (Z)-octadec-9-enoic acid (29 wt.%) and hexadecanoic acid (7 wt.%) in apple seeds [119]. Polyhydroxy-fatty acids, such as 9,10,18-trihydroxyoctadecanoic acid and 10,16-dihydroxyhexadecanoic acid, were reported to appear in apple-derived cutin in concentrations near 24 wt.%, in both cases [32]. (Z)-octadec-9-enoic acid (31 wt.%) was also determined in fresh apple seeds together with 9,10,18-trihydroxyoctadecanoic acid (20.32 wt.%) [120]. This latter compound was also found in cutin obtained from the golden and red apple varieties, accounting for 17.68 wt.% and 20.5 wt.%, respectively. Moreover, 10,16-dihydroxyhexadecanoic acid was also determined at concentrations of 15–19 wt.%, being (9Z,12Z)-octadeca-9,12-dienoic acid found in a lower content (1–2 wt.%) compared to that obtained in our work [37]. Leide et al. reported 10,16-dihydroxyhexadecanoic acid as the main monomer present in apple cutin, whereas 8-[3-(8-hydroxyoctyl)oxiran-2-yl]octanoic acid and 9,10,18-trihydroxyoctadecanoic acid were also highlighted as major cutin monomers [121]. These compounds also appeared in plants such as *Eucalyptus camaldulensis* and *Eucalyptus globulus,* with concentrations ranging from 14–22 wt.% for 9,10,18-trihydroxyoctadecanoicacid and from 17–28 wt.% for 10,16-dihydroxyhexadecanoic acid [122].

Differences found by other authors in monomers’ composition from the studied wastes could be related to the different varieties, state of ripening or geographic location of the raw materials, among others. In this work, it has been demonstrated that cutin obtained from tomato, watermelon and apple peels is a rich source of polyhydroxy fatty acids, such as 10,16-dihydroxyhexadecanoic acid in tomatoes and 9,10,18-trihydroxyoctadecanoic acid in apples. These compounds can be exploited as natural and renewable raw materials for the synthesis of polyhydroxyalkanoates with different applications, such as for the production of bioplastics. In addition, the cutin extracted from different tomato residues has been used for the development of films and coatings for cosmetics, biomedicine or food-packaging applications [32] Moreover, the cutin extracted from WP had a monomers’ composition mainly based on unsaturated fatty acids, which are known to be essential for human health and need to be supplied to the body through the diet. Fatty acids’ deficiency is normally associated with heart disease and high blood cholesterol levels. Watermelon cutin has also been demonstrated to be a novel and promising alternative source as a cutinase inducer [9]. In addition, these organic substances are known to be raw materials for the development of different products, such as biofuels, cosmetics, detergents and pharmaceuticals [123].

#### 3.3.4. Thermal Characterization

The thermal results obtained for the cutin samples by UAE are presented in Table 6. Three major degradation steps were found in all of the studied samples by TGA. The first step was associated with the decarboxylation and/or dehydration/esterification of the hydroxylated fatty acids and the presence of some hemicellulose in the sample which was related to non-hydrolysable cutin composition. After that, two consecutive steps were detected which were related to the complex matrix of the studied biomass samples, consisting of different mixtures of hydroxy acids. The final step with a higher degradation temperature was associated with the fragmentation of the aliphatic chain of fatty acids with a different long chain or their oligomers [13,124,125]. Tomato cutin showed significantly (*p* < 0.05) higher temperatures for the two first degradation steps compared to the other peel wastes. This behavior could indicate that the cutin derived from TP was composed of different monomers with a higher molecular weight. Regarding the cutin obtained from WP and AP, non-significant differences (*p* > 0.05) were observed in terms of degradation temperatures for these steps. The apple cutin showed a higher maximum degradation temperature for the third degradation step compared to the rest of the samples (*p* < 0.05) under optimal UAE conditions.

The DSC results showed the presence of two sharp peaks in tomato cutin around −19 °C and a broader one around 36 °C, which were attributed to the unsaturated and saturated fatty acids, respectively [13]. The second fusion step occurred in the same temperature range where the glass transition temperature was expected to be obtained, as previously reported [32]. For this reason, this transition was not observed after the second heating in the cutin from the TP waste. Regarding the cutin from the WP and AP, significant differences (*p* < 0.05) were obtained between the Tg values (36 ± 4 °C and 24 ± 4 °C for WP and AP, respectively), as the temperatures at which the segment motion of the macromolecules become thermally activated [32], which were associated with differences in the fatty acids’ composition, as previously discussed, and the possible bound-moisture producing a certain plasticizing effect on the cutin [11].

In conclusion, all of the samples showed an initial degradation temperature higher than 200 °C, in particular for TP; resulting in similar reported values for rubber and PE and higher than the other widely used polymers such as PVC, PS and PVA [39]. This high thermal stability could be attributed to the in situ three-dimensional and heavily cross-linked cutin structure [9]. As a result, the formulation of cutin-based biomaterials, using different scalable techniques, such as extrusion–injection, alone or combined with other polymer materials, could be a promising approach for the development of films or coatings due to the high barrier and hydrophobic properties of cutin [9,39,126,127].

## 4. Conclusions

In this work, a cascade biorefinery approach based on two sequential UAE processes was successfully applied to tomato, watermelon and apple peels to obtain proteins, antioxidant compounds and cutin, for the first time. The use of UAE offers great advantages at the industrial level due to its scalability potential and its alignment with the principles of sustainable development. The applied extraction steps led to a significant reduction in time and energy consumption compared to the other conventional methodologies. In addition, the proposed method has been demonstrated to be applicable to different waste sources using the same experimental conditions. This research work represents a milestone in the extraction of active compounds derived from agro-industrial wastes, showing the active fractions obtained have a high potential to be used for different applications, such as food packaging, cosmetic, medical or functional foods. Cutin characterization showed it to be rich in long-chain hydroxy fatty acids which could be innovative building-block chemicals for the synthesis of novel biomaterials and coatings. Moreover, the approach followed can contribute to a circular economy model for the tomato, apple and watermelon processing industries in compliance with the goals of the 2030 Agenda for sustainable development.

## Figures and Tables

**Table 1:** Composition of the different fractions obtained in the cascade extraction process from different peel wastes (*n* = 3, mean ±SD). Different superscripts in the same column indicate significantly different values (*p* < 0.05). YP, YAx and YC are the extraction yields of proteins, antioxidant compounds and cutin, respectively.

Waste	YP (wt.%)	Protein Content (mg BSA/g Extract)	YAx (wt.%)	YC (wt.%)	Non-Hydrolysable Cutin * (wt.%)
TP	9 ± 1 ^a^	590 ± 3 ^a^	36 ± 5 ^a^	14 ± 2 ^a^	42 ± 4 ^ab^
WP	7 ± 2 ^ab^	857 ± 1 ^b^	25 ± 6 ^b^	7 ± 1 ^b^	55 ± 9 ^a^
AP	5 ± 1 ^b^	625 ± 2 ^c^	32 ± 3 ^ab^	20 ± 1 ^c^	40 ± 6 ^b^

* Based on total content of cutin fraction.

**Table 2:** Total phenolic content and antioxidant activity of different extracts obtained from tomato, watermelon and apple wastes by UAE (*n* = 3, mean ±SD). Different superscripts in the same column indicate significantly different values (*p* < 0.05). dm: dry matter.

Waste	TPC (mg GAE/100 g dm)	ABTS (µmol TE/100 g dm)	FRAP (µmol TE/100 g dm)	DPPH (µmol TE/100 g dm)
TP	103.5 ± 0.9 ^a^	279 ± 3 ^a^	264 ± 2 ^a^	159 ± 1 ^a^
WP	107.2 ± 0.2 ^b^	356 ± 1 ^b^	507 ± 4 ^b^	158 ± 1 ^a^
AP	61.4 ± 0.1 ^c^	1559 ± 20 ^c^	1767 ± 5 ^c^	902 ± 16 ^b^

**Table 3:** UAE results obtained for Plackett–Burman (III) screening design in TP.

Run	EtOH (wt.%)	Energy (kWs)	Amplitude (%)	Ratio (mL/g)	pH	Acid	Yield (wt.%)
1	40	20	100	80	5.5	Hydrochloric	1.2
2	40	60	100	80	2.5	Citric	14.3
3	40	20	60	40	2.5	Hydrochloric	0.0
4	100	60	60	80	5.5	Hydrochloric	0.7
5	100	20	100	40	2.5	Hydrochloric	2.5
6	40	20	60	80	5.5	Citric	2.4
7	40	60	60	40	2.5	Citric	10.3
8	100	20	60	40	5.5	Citric	7.4
9	100	60	60	80	2.5	Hydrochloric	4.6
10	40	60	100	40	5.5	Hydrochloric	0.0
11	100	20	100	80	2.5	Citric	4.9
12	100	20	100	40	5.5	Citric	5.3

**Table 4:** Composition of cutin monomers obtained from the studied peel wastes, expressed as percentage of total peak areas in the GC/MS chromatogram (*n* = 3, mean ± SD).

Compound	TP		WP		AP	
	Quality	wt.%	Quality	wt.%	Quality	wt.%
Tetradecanoic acid	96	0.40 ± 0.10	98	0.55 ± 0.02	-	-
Hexadecanoic acid	96	13.02 ± 2.05	99	43.00 ± 1.02	99	8.45 ± 0.21
(Z)-hexadec-9-enoic acid	90	0.20 ± 0.05	-	-	-	-
(*E*)-hexadec-9-enoic acid	46	0.23 ± 0.04	86	1.00 ± 0.61	-	-
Heptadecanoic acid	98	0.13 ± 0.03	96	0.58 ± 0.01	-	-
(9Z,12Z)-octadeca-9,12-dienoic acid	96	9.01 ± 2.02	99	11.10 ± 0.41	96	20.02 ± 1.23
(Z)-octadec-9-enoic acid	96	5.02 ± 1.00	-	-	-	-
(E)-octadec-9-enoic acid	-	-	-	-	99	12.17 ± 0.49
Octadecanoic acid	99	3.00 ± 1.10	99	12.02 ± 1.02	99	4.52 ± 0.31
Icosanoic acid	96	1.21 ± 0.74	-	-	97	2.22 ± 0.14
Docosanoic acid	-	-			96	1.39 ± 0.18
10,16-dihydroxyhexadecanoic acid	46	59.00 ± 7.02	-	-	60	6.64 ± 0.33
9,10-Dihydroxyoctadecanedioic acid	-	-	43	2.51 ± 0.60	43	3.18 ± 0.11
9,10,18-trihydroxyoctadecanoic acid	87	0.61 ± 0.04	-	-	90	10.53 ± 0.61
(3S)-3-methyl-2-oxopentanoic acid	47	0.32 ± 0.13	-	-	-	-
(9Z,12Z,15Z)-octadeca-9,12,15-trienoic acid	-	-	99	16.00 ± 0.52	53	4.02 ± 2.00
(2E,4E)-octadeca-2,4-dienoic acid			-	-	42	1.31 ± 0.24
Pentan-2-ol	-	-	41	0.55 ± 0.03	-	-
(E,7R,11R)-3,7,11,15-tetramethylhexadec-2-en-1-ol	-	-	97	0.80 ± 0.12	-	-
[(Z)-octadec-9-enyl] formate	-	-	-	-	78	1.00 ± 0.12
8-[3-(8-hydroxyoctyl)oxiran-2-yl]octanoic acid	-	-	-	-	53	1.72 ± 0.35

**Table 5:** Main cutin monomers obtained from tomato, watermelon and apple peels, expressed as percentage of total peak areas in the GC/MS chromatogram, and comparison with data reported in literature. Mean ± SD (wt.%).

Compound.	TP		WP		AP	
	This work	[11]	This work	[117]	This work	[37]
Hexadecanoic acid	13.02 ± 2.05	1–2	43.00 ± 1.02	28.42 ± 3.30	8.45 ± 0.21	1–2
(9Z,12Z)-octadeca-9,12-dienoic acid	9.01 ± 2.02	2–4	11.10 ± 0.41	15.86 ± 0.77	20.02 ± 1.23	1
(E)-octadec-9-enoic acid	-	-	-	-	12.17 ± 0.49	-
(Z)-octadec-9-enoic acid	5.02 ± 1.00	-	-	-	-	-
(9Z,12Z,15Z)-octadeca-9,12,15-trienoic acid	-	-	16.00 ± 0.52	-	4.02 ± 2.00	-
Octadecanoic acid	3.00 ± 1.10	0.8	12.02 ± 1.02	-	4.52 ± 0.31	-
9,10-Dihydroxyoctadecanedioic acid	-	-	2.51 ± 0.60	-	3.18 ± 0.11	-
10,16-dihydroxyhexadecanoic acid	59.00 ± 7.02	62–82	-	-	6.64 ± 0.33	15–19
9,10,18-trihydroxyoctadecanoic acid	0.61 ± 0.04	-	-	-	10.53 ± 0.61	18–21

**Table 6:** Thermal results obtained for cutin derived from different peel wastes (*n* = 3) Means ±SD. Different superscripts in the same column indicate significantly different values (*p* < 0.05).

Waste	T_max1_ (°C)	T_max2_ (°C)	T_max3_ (°C)	Solid residue (wt.%)	T_g_ (°C)
TP	301 ± 3 ^a^	372 ± 2 ^a^	445 ± 1 ^a^	19 ± 1 ^a^	-
WP	199 ± 2 ^b^	306 ± 2 ^b^	447 ± 1 ^a^	18 ± 1 ^a^	36 ± 4 ^a^
AP	203 ± 2 ^b^	302 ± 2 ^b^	471 ± 3 ^b^	18 ± 1 ^a^	24 ± 4 ^b^

## Data Availability

Data are contained within the article.
